# Supplementary material for: Rare CIDEC coding variants enriched in age-related macular degeneration patients with small low-luminance deficit cause lipid droplet and fat storage defects
Source: PLoS One. 2023 Apr 20;18(4):e0280484. doi: 10.1371/journal.pone.0280484 (PMC10118094; doi:10.1371/journal.pone.0280484)
Supplement: S1 File — (PDF) [file pone.0280484.s001.pdf]

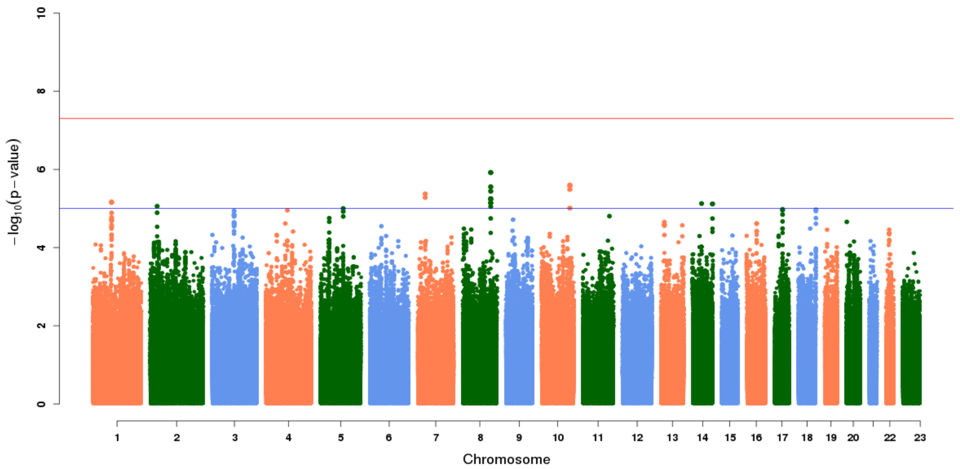

S1 Fig: Manhattan plot of common variant analysis results contrasting AMD patients in the top and bottom LDD quartiles (Q1 and Q4).

In adipose tissue, only 0.5% of CIDEC transcripts contain exon 4

CIDEC transcripts (RefSeq)

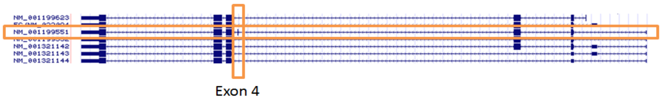

RefSeq transcript	Exon 4	% expression (transcripts per million)
NM_001199623		12.8%
NM_022094		4.5%
**NM_001199551**	✔	**0.5%**
NM_001199552		49.7%
NM_001321142		21.1%
NM_001321143		5.0%
NM_001321144		6.4%

159 adipose tissue donors

Source: GTEx, Salmon Isoform Quantification

S2 Fig: Summary of CIDEC exon expression in adipose tissue. *CIDEC* exon 4, the location of all rare variants seen in Q4 AMD patients, is expressed in 0.5% of all CIDEC transcripts found in adipose tissue in samples from the GTEx project.

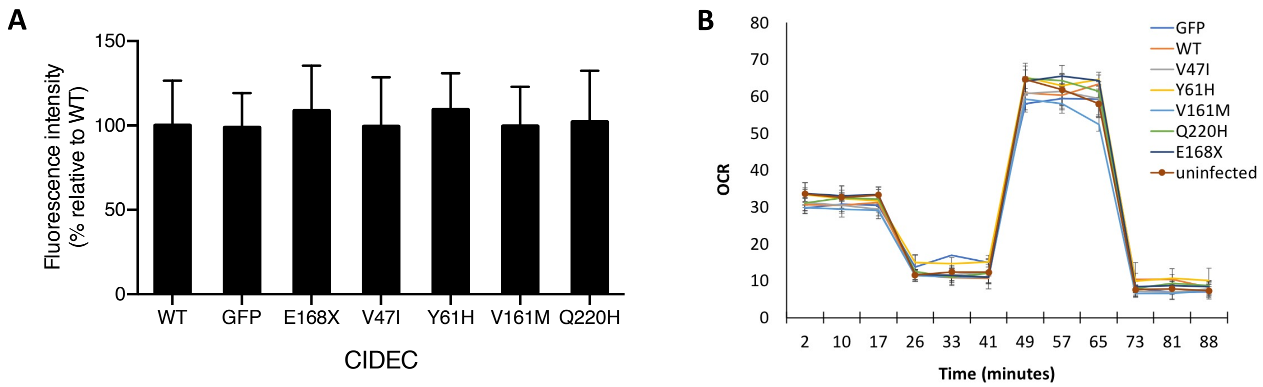

S3 Fig: CIDEC rare variants do not affect mitochondria density or function. Quantification of mitochondria density using MitoTracker in 3T3-L1 cells expressing CIDEC wild-type (WT) or each of the rare variants (A). Mitochondria function measured by Seahorse analyzer (OCR: Oxygen consumption rate) (B).

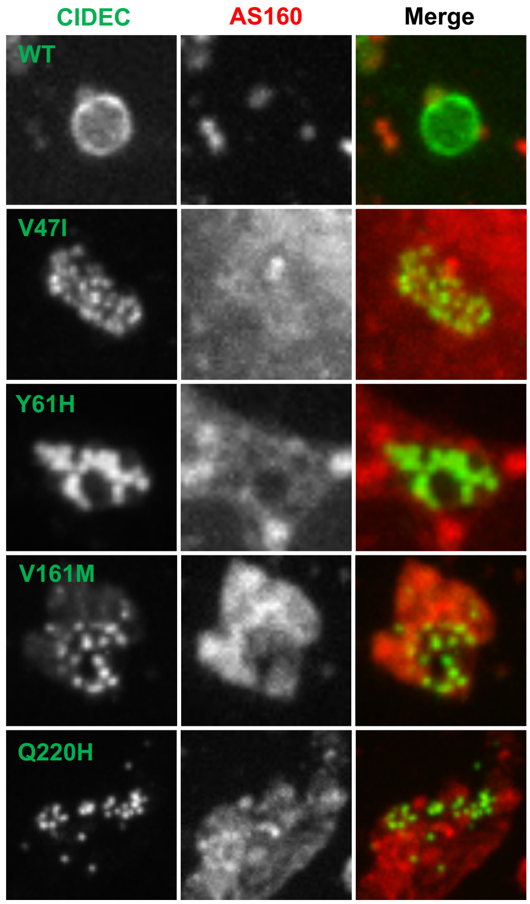

S4 Fig: Representative images of CIDEC wild-type (WT) or rare variants (green) and AS160 (red) in pre-adipocytes.

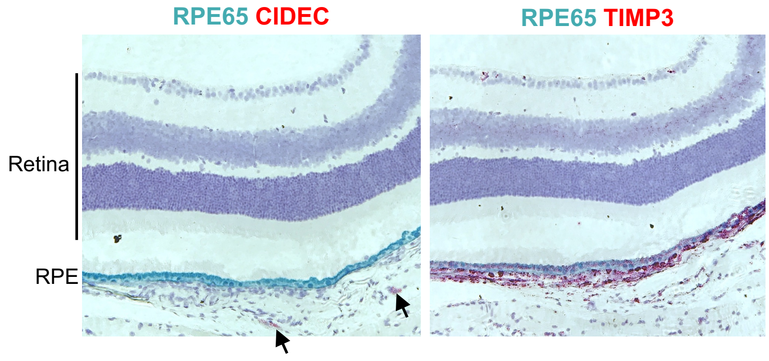

S5 Fig: By in situ hybridization (ISH), Cidec RNA is not detected in mouse retina and Retinal Pigment Epithelium (RPE) cells. Rare Cidec positive cells are present in the choroidal tissue underneath the RPE (left: red, arrows). ISH for Rpe65 was used as RPE cell marker, and ISH for Timp3 (right: red) was used as positive control.

SNP	LOCUS	CHR	BP	A1	Q1 MAF	Q4 MAF	OR	p value
rs10033900	*CFI*	4	109737911	T	0.47	0.52	0.83	0.20
rs10781182	*MIR6130-RORB*	9	74002804	T	0.36	0.36	0.94	0.69
rs10922109	*CFH*	1	196735502	A	0.26	0.23	1.10	0.47
rs11080055	*TMEM97-VTN*	17	28322698	A	0.45	0.47	0.91	0.49
rs114092250	*PRLR-SPEF2*	5	35494346	A	0.02	0.03	0.78	0.58
rs1142	*KMT2E-SRPK2*	7	105115879	T	0.38	0.33	1.24	0.13
rs12357257	*ARHGAP21*	10	24710664	A	0.20	0.29	0.63	0.004
rs140647181	*COL8A1*	3	99461824	C	0.03	0.03	1.01	0.97
rs1626340	*TGFBR1*	9	99161090	A	0.17	0.20	0.90	0.54
rs2043085	*LIPC*	15	58388755	T	0.40	0.35	1.29	0.10
rs2230199	*C3*	19	6718376	C	0.28	0.27	0.99	0.96
rs3138141	*RDH5-CD63*	12	55721994	A	0.20	0.25	0.79	0.17
rs3750846	*ARMS2-HTRA1*	10	122456049	C	0.41	0.41	1.05	0.71
rs429358	*APOE*	19	44908684	C	0.09	0.11	0.89	0.62
rs5754227	*SYN3-TIMP3*	22	32709831	C	0.10	0.10	1.03	0.89
rs61941274	*ACAD10*	12	111694806	A	0.03	0.04	0.75	0.48
rs61985136	*RAD51B*	14	68302482	C	0.34	0.36	0.94	0.68
rs62247658	*ADAMTS9-AS2*	3	64729479	C	0.45	0.50	0.83	0.18
rs67538026	*CNN2*	19	1031439	T	0.46	0.48	0.93	0.62
rs72802342	*CTRB2-CTRB1*	16	75200974	A	0.07	0.06	1.34	0.32
rs7803454	*PILRB-PILRA*	7	100393925	T	0.21	0.20	1.25	0.20
rs8135665	*SLC16A8*	22	38080269	T	0.21	0.20	1.04	0.83
rs943080	*VEGFA*	6	43858890	C	0.45	0.43	1.10	0.50
rs9564692	*B3GALTL*	13	31247103	T	0.28	0.25	1.21	0.22

S1 Table: Comparison of AMD associated risk variants from Fritsche et. al, Nat Gen, 2015 in Q1 and Q4 AMD patients.

	Q1	Q4	p value	Missing (N%)	
				Q1	Q4
N	171	170			
Age, mean (sd)	77.8 (7.9)	75.1 (8.1)	0.002	0 (0%)	0 (0%)
Female, N (%)	104 (60.1%)	113 (66.5%)	0.33	0 (0%)	0 (0%)
Baseline visual acuity, mean (sd)	42.0 (8.7)	74.2 (2.2)	<2E-16	0 (0%)	0 (0%)
Baseline CNV leakage area	7.6 (5.3)	6.1 (4.2)	0.05	104 (60.8%)	71 (71.7%)

S2 Table: Quartile Q1 and quartile Q4 AMD TENAYA and LUCERNE patient demographic comparison.
