# Supplementary figures and images for: Rare CIDEC coding variants enriched in age-related macular degeneration patients with small low-luminance deficit cause lipid droplet and fat storage defects
Source: PLoS One. 2023 Apr 20;18(4):e0280484. doi: 10.1371/journal.pone.0280484 (PMC10118094; doi:10.1371/journal.pone.0280484)

**A**

3xFlag-CIDEDEC

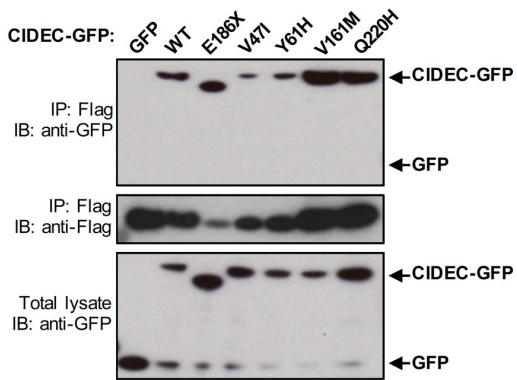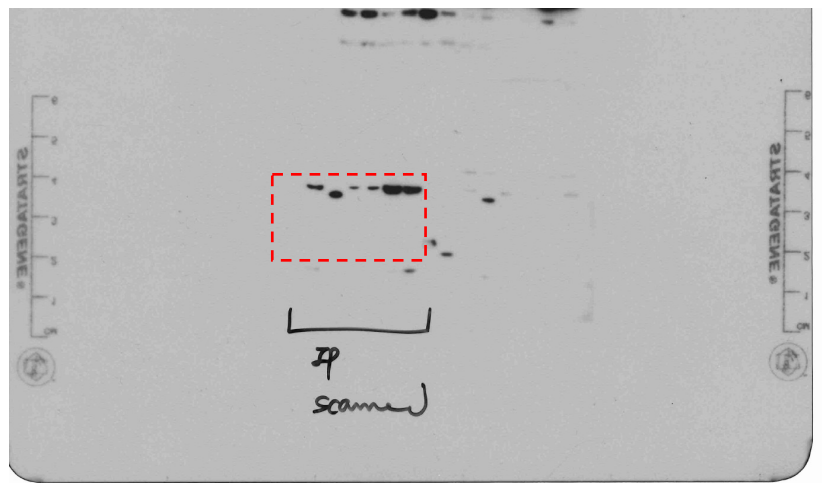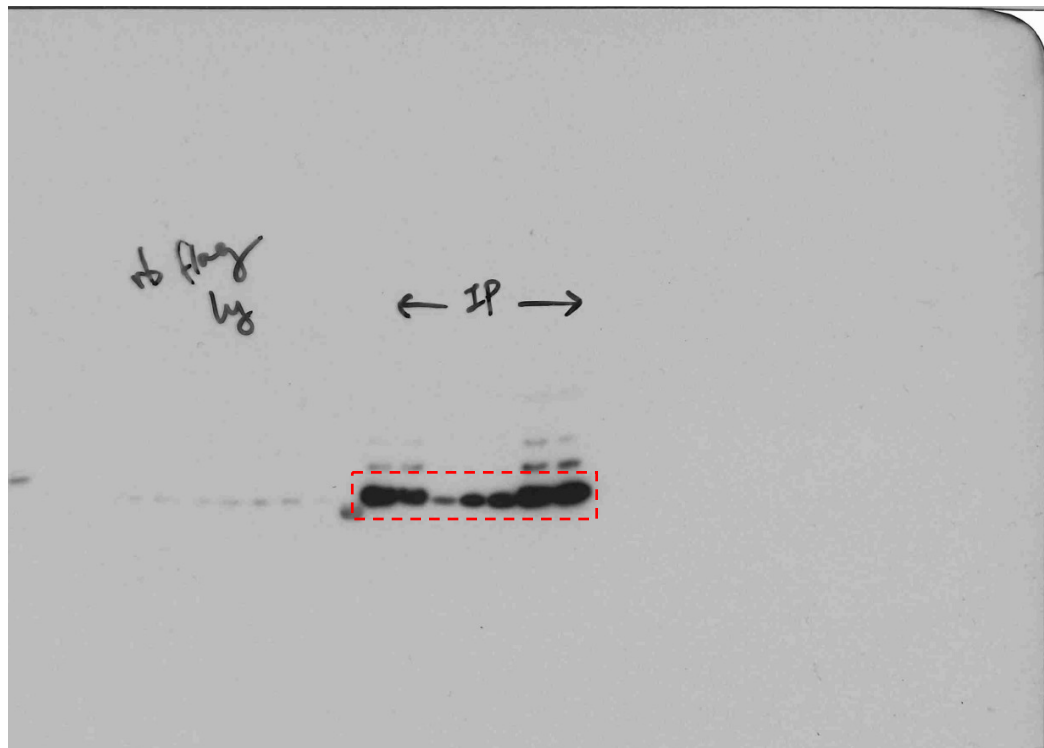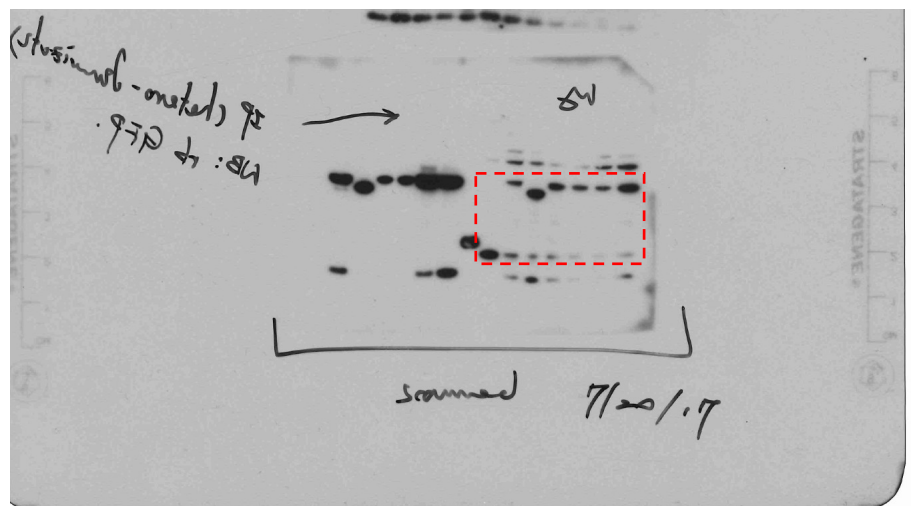

**B**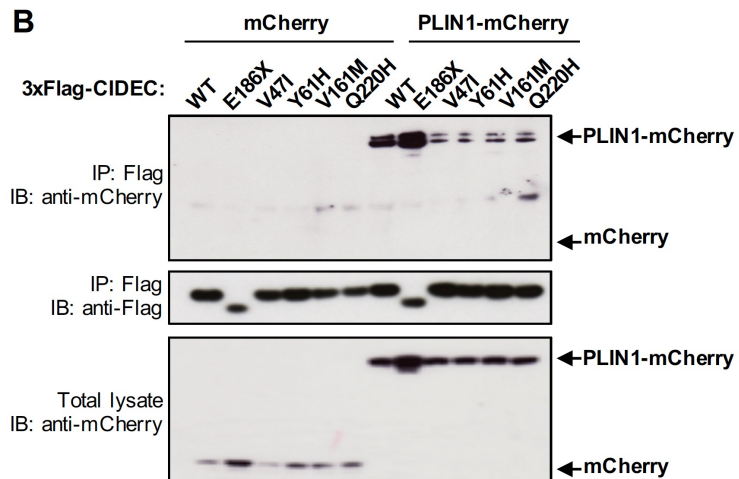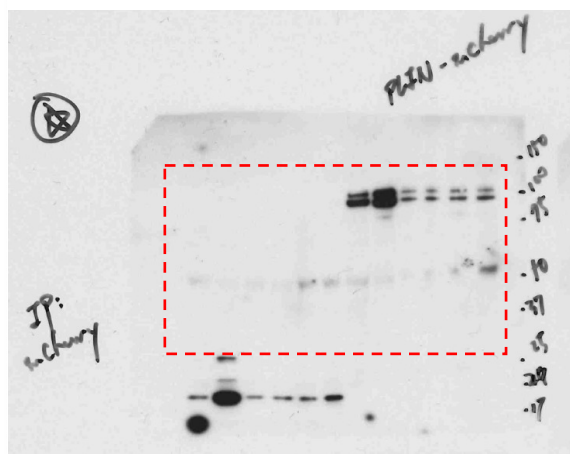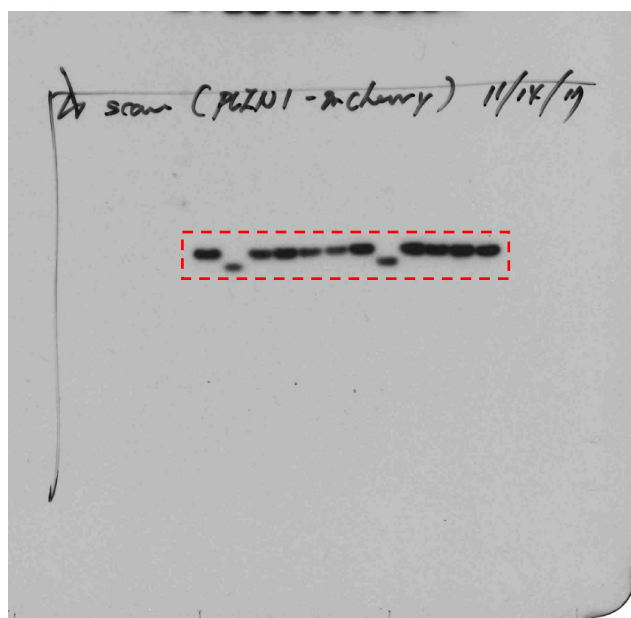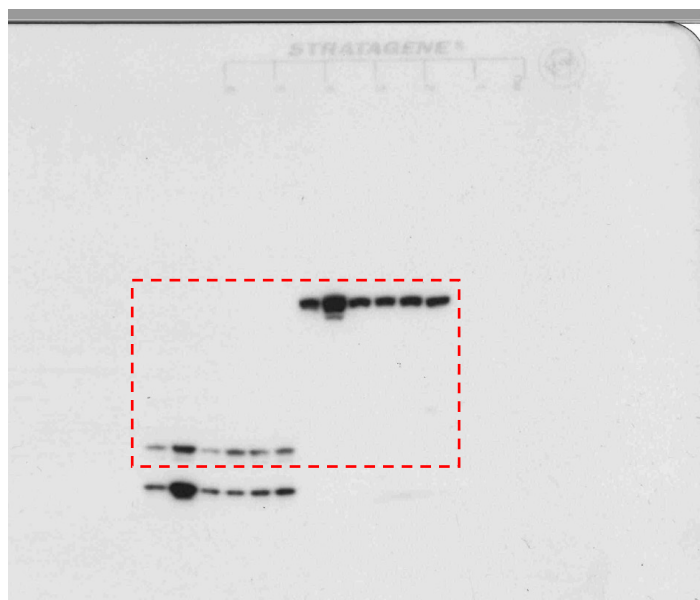

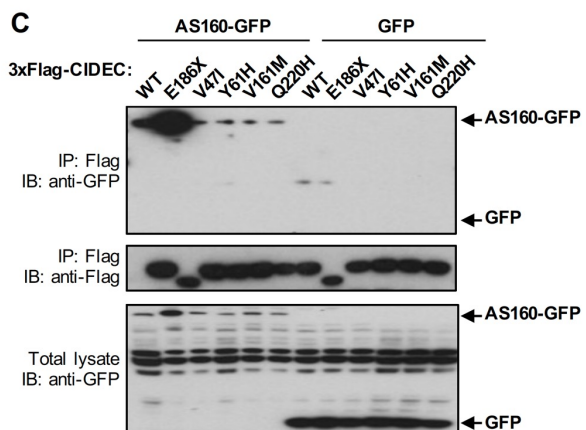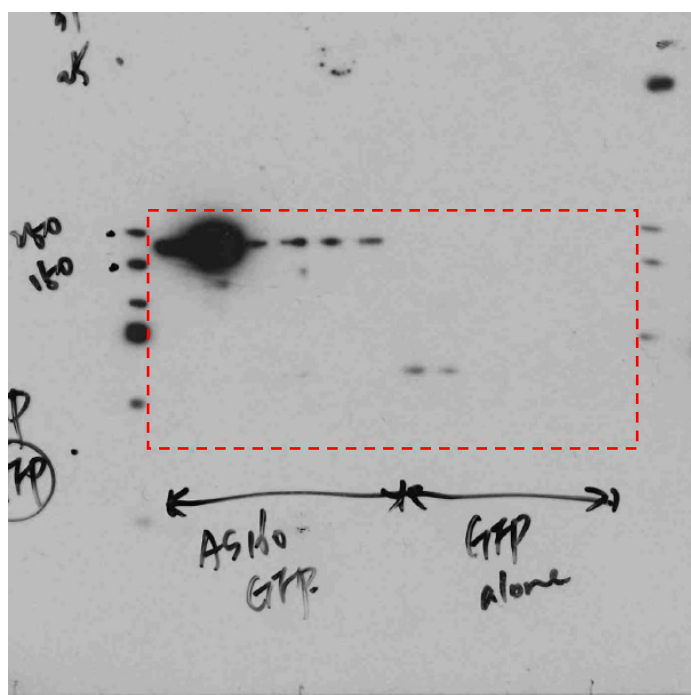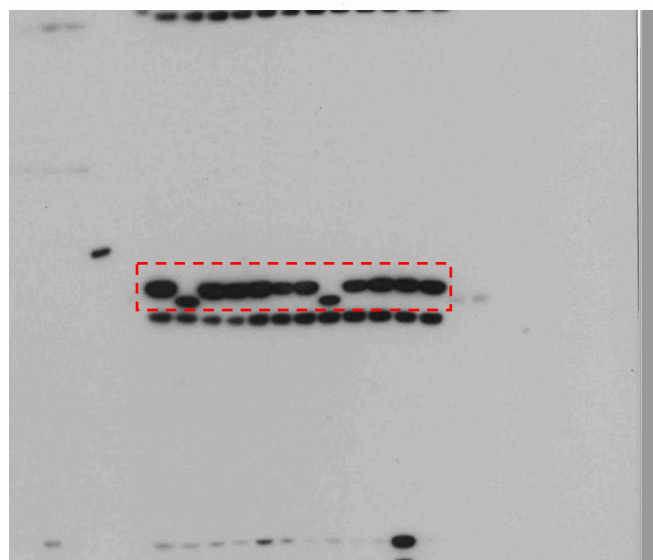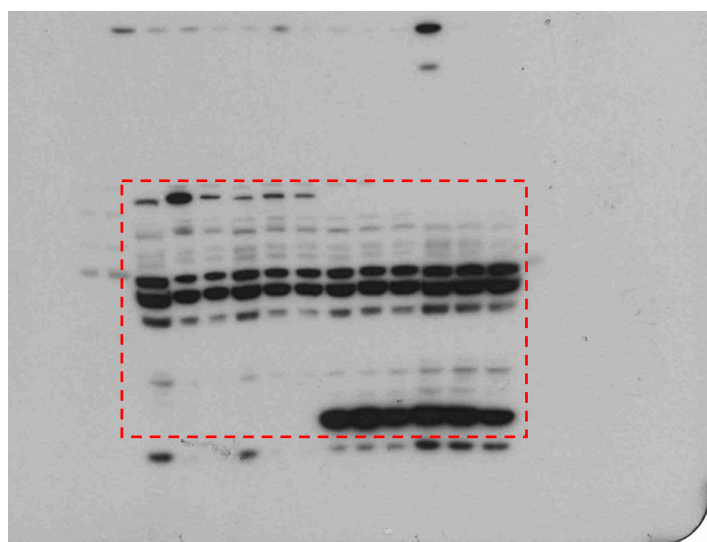

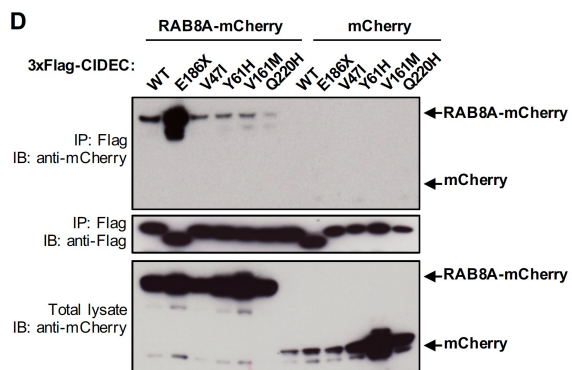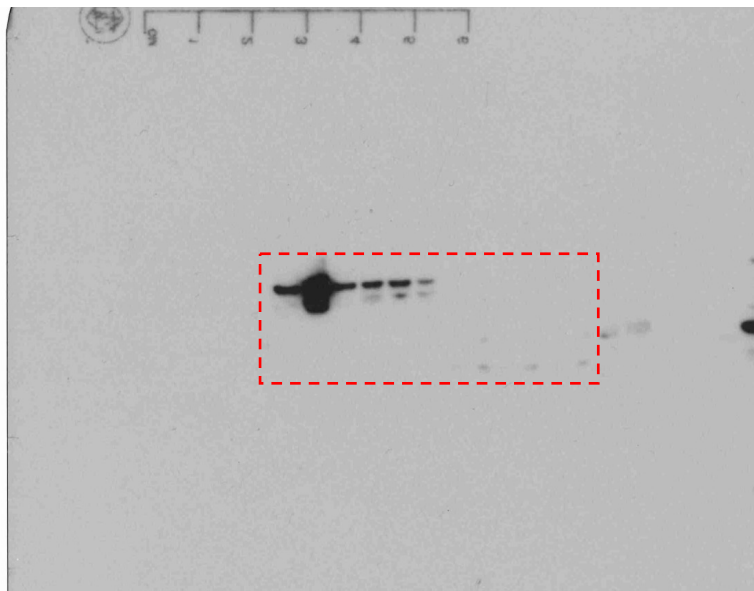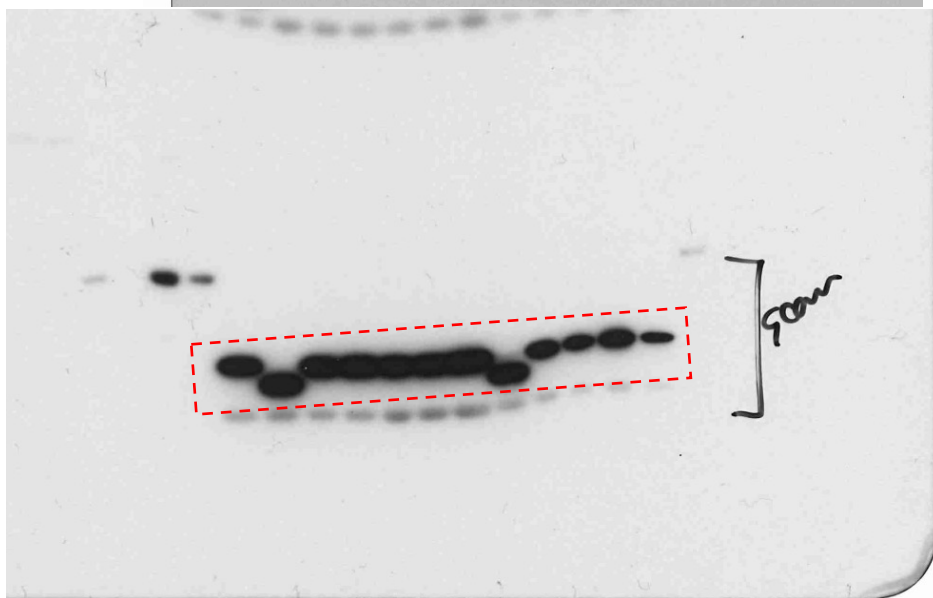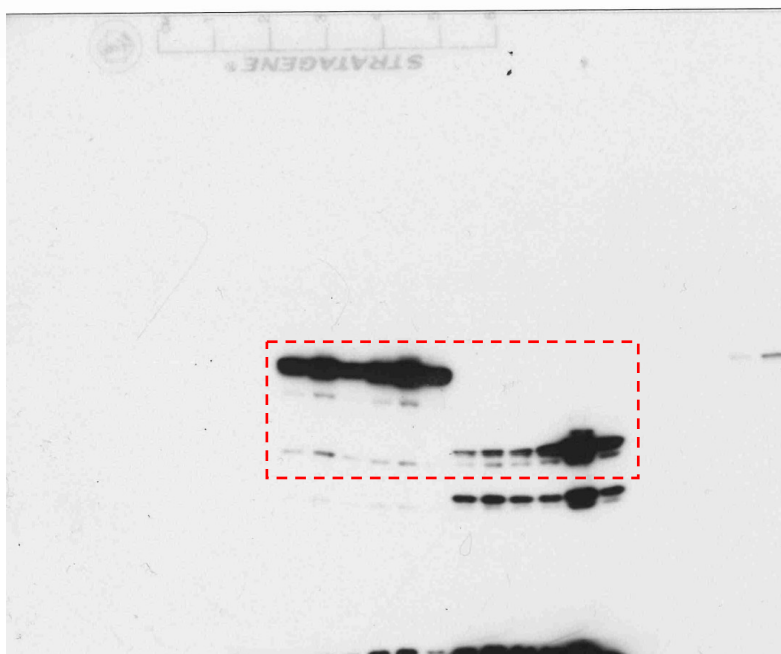

Supplement: S1 Raw images — The area used in Fig 6 are highlighted on each film by red rectangles. (PDF) [file pone.0280484.s002.pdf]

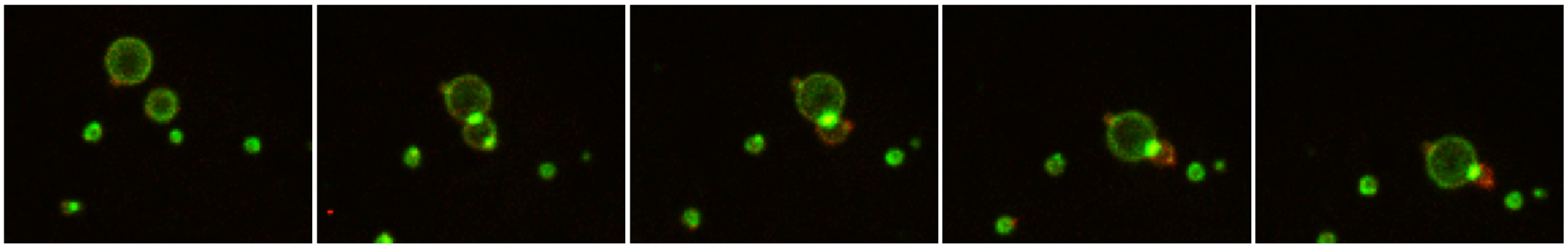

Supplement: S1 Fig — (PNG) [file pone.0280484.s004.png]
