# Supplementary material for: Rare CIDEC coding variants enriched in age-related macular degeneration patients with small low-luminance deficit cause lipid droplet and fat storage defects
Source: PLoS One. 2023 Apr 20;18(4):e0280484. doi: 10.1371/journal.pone.0280484 (PMC10118094; doi:10.1371/journal.pone.0280484)
Supplement: Attachment — Submitted filename: Response27-05-2022-RA-eLife-80645.docx [file pone.0280484.s005.docx]

Manuscript: **27-05-2022-RA-eLife-80645**

“Age-related Macular Degeneration patient deep phenotyping and whole genome sequencing analysis identifies coding variants linking small low-luminance visual deficit to fat storage defects”

The initial decision of not considering our manuscript further for publication was based on concerns related to the sample size and lack of genome-wide significant findings that would not be addressed without replication in an independent cohort. We are pleased to report that we have recently gained access to a second, independent patient cohort. While there are still no genome-wide significant findings, the CIDEC gene is now the top gene in the rare variant burden test meta-analysis of the discovery and replication cohorts (OR = 5.71, p = 4.1x10^-4^). The revised manuscript is greatly strengthened by the addition of these data. Indeed, both the genetic analysis and the ad-hoc prioritization now identified the same top gene, CIDEC, and support our detailed functional downstream analysis.

**Response to the Decision letter and Reviews:**

Dear Dr Jeanne,

Thank you for submitting the paper "Age-related Macular Degeneration patient deep phenotyping and whole genome sequencing analysis identifies coding variants linking small low-luminance visual deficit to fat storage defects" for consideration by eLife. Your article has been reviewed by 2 peer reviewers, and the evaluation has been overseen by a Reviewing Editor and a Senior Editor.

**Comments to the Authors:**

We are sorry to say that, after consultation with the reviewers, we have decided that this work will not be considered further for publication by eLife.

Both reviewers raised concerns related to the sample size and lack of genome-wide significant findings that form the basis of the functional work that would not be able to be addressed unless the overall study is repeated.

Yes, this is correct, we did not have genome-wide significant findings in our study. To address this we conducted a thorough follow-up analysis on one of the top genes in the genetic analysis. *Additionally, we have added to the manuscript results of an analysis for the top genes from the study in a second, independent cohort which has recently become available as a replication cohort.* While the results still do not reach genome-wide significance, CIDEC is the top gene in the rare variant burden test meta-analysis of the discovery and replication populations by p-value (p=4.10x10^-4^). We note that through both the replication and the ad-hoc prioritization approaches, we landed on the same gene, CIDEC, which is the focus of the in vitro work of the study. Of note, these are currently the only datasets worldwide we are aware of with whole genome sequencing and the deep patient phenotyping to conduct focused studies such as this. We do not think that interesting hits should be ignored until they reach a certain level of significance, but rather should be investigated if they are of strong biologic interest. Furthermore, the functional validation revealed that all the rare variants identified in the AMD Q1 patients have the same hypomorphic defect affecting some but not all CIDEC cellular functions, and that this defect is different than defects caused by another rare variant identified in a different patient population (lipodystrophy).

We think this is of use to report, given the dearth of AMD genetics studies focused on clinical factors of importance in disease progression and treatment response. We look forward to others being able to confirm our genetic hypotheses in additional datasets when similar studies are conducted.  

**Public Evaluation Summary:**

In this study, the investigators assess anti-VEGF (ranibizumab) treatment response in age-related macular degeneration (AMD) using a biomarker for this response, low-luminance vision deficit (LLD). The authors then performed a variety of genetic association analyses: (i) AMD risk variant analysis, (ii) polygenic risk (AMD and related traits) (iii) GWAS (common variants), and (iv) gene burden analysis (rare variants). The strength of this study is the in-vitro validations provided following in-silico findings, which is uncommon in GWAS studies.

**Reviewer #1 (Public Review):**

In this study, the investigators assess anti-VEGF (ranibizumab) treatment response in age-related macular degeneration (AMD) using a biomarker for this response, low-luminance vision deficit (LLD).

The recruited individuals were extensively phenotyped in the HARBOR clinical trial (n=1098) and individuals at the ends of the LLD underwent whole genome sequencing, as follows:

Quartile 1: smallest LLD (responders, n=275)

Quartile 4: largest LLD (non-responders, n=241)

The authors then performed a variety of genetic association analyses: (i) AMD risk variant analysis, (ii) polygenic risk (AMD and related traits) (iii) GWAS (common variants), and (iv) gene burden analysis (rare variants).

Major weaknesses:

None of the association results survived correction for multiple testing and no power calculations are mentioned. Correction for multiple testing is standard in the field of human genetics and has helped address prior replication issues with genetic associations and human traits.

We refer the editor to the above paragraph referencing the additional study population added to the manuscript.

The authors then chose to focus on one of the 'top ranked' rare analysis genes (uncorrected P=0.001). All results are based on a maximum of 12 carriers of CIDEC rare variants (four unique variants). The genetic association results therefore do not warrant functional validation at this time.

We respectfully disagree with this statement. While the results are not genome-wide significant, that does not mean they are not worthy of follow-up. In this study we found rare, functionally predicted variants to be enriched in patients with better outcome and response to treatment for macular degeneration. From a drug discovery standpoint, this is quite interesting, as it implies knockdown would be beneficial to patients. As such, it is deserving of *in vitro* follow-up, especially when sizable datasets do not exist for further examination of the genetic finding.

The functional genomics experiments fall outside my current area of expertise but appear to be extensive. However, this does not circumvent the fact that they are based on weak genetic evidence. In the human genome, rare variants are enriched for deleterious variants - validating that rare variants influence gene function does not provide validation of genetic association results.

We have added additional genetic data which enhance the choice of CIDEC as a candidate gene for follow-up. That, along with the prioritization process we developed, both pointed to CIDEC being of interest.

**Reviewer #1 (Recommendations for the authors):**

Due to the issues related to power and multiple testing correction, these preliminary results may be more suited to a specialist journal. The title should be rephrased to reflect this, removing "identifies" with a more appropriate verb.

We thank the reviewer for this suggestion. Our initial title was: “Rare CIDEC coding variants enriched in Age-related Macular Degeneration patients with small low-luminance deficit cause lipid droplet and fat storage defects”. We have been asked to edit the title to fit better to the journal format guidelines but would be happy to switch back to the original title.

The reference used for justification of extreme phenotyping selection (i.e., ref 15) states the following: "For studies of quantitative traits, it has been shown that the power to detect rare variant associations can be increased by sampling from the extremes of the trait distribution [27,28]. To do so, typically the phenotype (or a transformed version of the phenotype) is assumed to follow a normal distribution. Then, the largest and smallest n th percentile of the distribution are chosen for study, where n is typically less than five." The current approach of taking quartiles goes against this guidance. A quantitative approach with the entire cohort would therefore probably be more appropriate.

For this study, the reason to select the extremes was based off of previous work done on the patients in this same study. We refer the reviewer to Frenkel, et al, British Journal of Ophthalmology 2016, figure 2 (pasted below) where there is clear delineation between Q1 and Q4 with regards to treatment response, with Q2 and Q3 overlapping between them. This indicates the real power to detect an association with this phenotype would lie in the extremes and the middle quartiles are not informative. This matches with the phenotype where patients may read better or worse on a given day, which would make the middle area a bit ambiguous. To test this, we looked at CIDEC significance with LLD as a continuous outcome, finding a P=0.53, b=-0.08 for CIDEC.

Minor revisions:

Abstract: Please add sample size of cohort and P-values

Introduction: Please update variants to loci in the following statement: "52 independently associated variants"
Metrics should be provided related to the utility of LLD as a biomarker for AMD (e.g., sensitivity, specificity, etc.)

Line 166: What direction is the "eye problems/disorders" association for CIDEC in UKB GENEBASS?

There is no information regarding the processing of sequence reads and variant calling in the main manuscript. Please add this.

The data sharing policy should be revised as it does not follow current best practices in human genomics. It currently states: "...the summary statistics for the genetics analysis can be provided upon request to the corresponding author (Dr Marion Jeanne: [jeanne.marion@gene.com](mailto:jeanne.marion@gene.com)) and/or the lead Human Geneticist (Dr Brian Yaspan: [yaspan.brian@gene.com](mailto:yaspan.brian@gene.com)). Data is available for qualified researchers employed or legitimately affiliated with an academic, non-profit or government institution who have a track record in the field."

While it is reasonable not to share individual-level genetic data, sharing summary statistics in public repositories (e.g., GWAS Catalog) is now standard and expected for GWAS.

While this would be ideal, the patient consent forms for these studies and the vendor contract for sequencing do not allow depositing of summary statistics in online databases such as the GWAS catalog. We are happy to provide summary statistics to those interested should they contact us.

**Reviewer #2 (Public Review):**

The authors conducted the first genome-wide association study (GWAS) of low-luminance vision deficit followed by several in-vitro validation experiments. The strength of this study is that the authors provide in-vitro validations following in-silico findings, since most GWAS only include computational results. However, the weakness is that the in-silico findings are not significant, which make the downstream in-vitro validations less convincing.

We refer the reviewer to the statement to the editor regarding the non-genome wide significant genetic findings

**Reviewer #2 (Recommendations for the authors):**

My major concern is why authors performed such in-vitro validations given the insignificant in-silico findings? I have the following specific questions.

1. Initially, sample size is ~1000. The outcome is continuous LLD. Instead of treating LLD as a continuous outcome. Authors dichotomized it using Q1 vs. Q4. So, only ~500 samples left in the GWAS analysis. Authors claimed that this is a way to increase power in genetic studies. This claim is counterintuitive. Since genetic effects are generally small, people usually improve power by increasing sample size. Also, continuous outcomes often have better power than binary outcomes, if there are true effects, although this may not be true for all traits. The citation used to support this claim is for "rare variant association", not genetic study in general. Even for rare variants, this may not be convincing, because the rare variants become even rarer by losing half samples. More importantly, authors didn't pick up any genome-wide significant common or rare loci. I wonder what the results would be if using all samples and treating LLD as continuous.

We refer the reviewer to the response of the first point made by reviewer 1.

2. In Figure 1, authors show gene expression of the top genes. What is the sample size for gene expression. Is that possible to conduct eQTL?

Gene expression data shown in Figure 1 is based on 96 Retinas and 106 RPE/Choroid samples. Given the rarity of the CIDEC SNPs in the study, it is not possible to conduct an eQTL analysis.

3. Authors may consider excluding the PRS analysis. IAMDGC (no AMD vs. AMD) used as the base data providing summary statistics is different from this current study (small LLD vs. large LLD within neovascular AMD). They are not comparable. Not surprising that the results are not significant. Also, "After quality control procedure, 24 SNPs..." is vague, no idea what has been done to get the 24 SNPs.

We thank the reviewer for this suggestion. As this is the first investigation of this trait, it is useful to see if risk genetics data can predict LLD at baseline as it is currently a topic in the field if risk genetics is useful for predicting patient outcome measures. In this instance, it is not. Quality control procedures are described in the methods as indicated in point 6. The statement "After quality control procedures" has been removed from the text in question, so the statement now reads "Twenty four SNPs..."

4. In Table 2, some explanation is needed in the footnote for Q1 freq, Q4 freq and OR, since there are multiple SNPs in each gene. It looks that a "new" SNP is created by adding up those SNPs, simple sum-up or weighted sum-up?

As this is a burden test, we are reporting the fraction of individuals in the population that have any rare, functionally predicted variant. The following text has been added to Table 2: “Frequencies in the columns denote the fraction of individuals in that cohort who possess any rare, functionally predicted variant identified in the study.”

5. Still in Table 2, are those rare SNPs only from exons or both exons and introns? In burden test, do you focus on exon SNPs? Since authors use WGS, may consider taking advantage of rare variants outside exons, otherwise WES would be enough. In the later analysis for CIDEC, these 5 rare SNPs seem from exons only. Exon SNPs are definitely more important, because they directly change downstream RNA. However, in this study, CIDEC doesn't express. It's hard to explain how the exon variants affect downstream changes. Can authors discuss more about this?

Yes, the SNPs are all exonic in table 2. Also yes, we focus on exonic SNPs only for the burden test.

As described in the methods section, exonic SNPs with a MAF<1% were required to be included in the analysis. While we agree with the reviewer's suggestion to take advantage of rare variants outside of exons would be desirable, we are currently unaware of a suitable method to analyze them methodically on a genome-wide scale.

While CIDEC doesn't express in ocular tissue, it is still expressed in humans and is a circulating factor, leading to our thought that once patients have developed advanced AMD, the disease outcome could be modified by systemic and indirect lipidomic biological processes. This is mentioned in the discussion.

6. Authors use MAF<0.01 as rare variant cutoff. I'm wondering how many of the rare variants have only one minor allele (minor allele counts)? If sizable rare variants have only one or two minor alleles, authors need to discuss how to ensure they are not due to technical errors (e.g., the depth of reads covers these rare variants).

We have added the following to the methods section to discuss read depth and quality control procedures:

The WGS data for the discovery study population was generated to a read depth of 30X using the HiSeq platform (Illumina X10, San Diego, CA, USA) processed using the Burrows-Wheeler Aligner (BWA) / Genome Analysis Toolkit (GATK) best practices pipeline. WGS short reads were mapped to hg38 / GRCh38 (GCA_000001405.15), including alternate assemblies, using BWA version 0.7.9a-r786 to generate BAM files. All WGS data was subject to quality control and checked for concordance with SNP fingerprint data collected before sequencing. After filtering for genotypes with a GATK genotype quality greater than 90, samples with heterozygote concordance with SNP chip data of less than 75% were removed. Sample contamination was determined with VerifyBamID software, and samples with a freemix parameter of more than 0.03 were excluded. Joint variant calling was done using the GATK best practices joint genotyping pipeline to generate a single variant call format (VCF) file. The called variants were then processed using ASDPEx to filter out spurious variant calls in the alternate regions.
Sample genotypes were set to missing if the Genotype Quality score was less than 20 and SNPs were removed if the missingness was higher than 5%. SNPs were filtered if the significance level for the Hardy-Weinberg equilibrium test was less than 5x10-8. The allele depth balance test was performed to test for equal allele depth at heterozygote carriers using a binomial test (discovery WGS only); SNPs were excluded if the p-value was less than 1x10-5.

7. No details about WGS in Materials and Methods, such as read depth, missingness, genotype quality, hwe, etc.

We refer the reviewer to point 6

Minor comments:

1. Authors claimed that clinical and genetic knowledge for AMD progression is not well understood. I suggest authors to do some literature review on AREDS and AREDS2, which are longitudinal studies initiated by NEI. AMD progression has been studied in several articles.

Yes, we agree with the reviewer that there is extensive literature regarding clinical knowledge of AMD progression markers. The point of the statement was regarding biomarkers that are of use in clinical trials, however, we agree it is an awkward statement as written. We have removed this sentence from the manuscript.

2. Authors seem to use neovascular AMD and advanced AMD interchangeably. It's better to consistently use neovascular AMD, because this study doesn't consider GA.

We thank the reviewer for pointing this out. We have replaced “advanced AMD” by “neovascular AMD” when appropriate.

3. In Figure 1, there seems to be a typo, it should be "rare variants", not "rave variants".

The typo in figure 1 legend has been corrected.

4. What is the Bonferroni adjusted cutoff for rare variant tests?

The Bonferroni cutoff has been added to the methods section (P<3.83x10-6). While we stated the number of genes tested, we apologize for the omission of the Bonferroni correct P.
